# Genetic differentiation and phylogeography of rotifer *Polyarthra dolichoptera* and *P. vulgaris* populations between Southeastern China and eastern North America: High intercontinental differences

**DOI:** 10.1002/ece3.8912

**Published:** 2022-05-13

**Authors:** Diwen Liang, George B. McManus, Qing Wang, Xian Sun, Zhiwei Liu, Senjie Lin, Yufeng Yang

**Affiliations:** ^1^ 47885 Department of Ecology Key Laboratory of Philosophy and Social Science in Guangdong Province Southern Marine Science and Engineering Guangdong Laboratory (Zhuhai) Jinan University Guangzhou P. R. China; ^2^ State Environmental Protection Key Laboratory of Urban Ecological Simulation and Protection South China Institute of Environmental Sciences MEE Guangzhou China; ^3^ 7712 Department of Marine Sciences University of Connecticut Groton Connecticut USA; ^4^ 26469 School of Marine Science Sun Yat‐Sen University Guangzhou P. R. China

**Keywords:** cryptic species, genetic distance, long‐distance dispersal, phylogeography, Rotifer, spatio‐temporal pattern

## Abstract

Genetic differentiations and phylogeographical patterns of small organisms may be shaped by spatial isolation, environmental gradients, and gene flow. However, knowledge about genetic differentiation of rotifers at the intercontinental scale is still limited. *Polyarthra dolichoptera* and *P*. *vulgaris* are cosmopolitan rotifers that are tolerant to environmental changes, offering an excellent model to address the research gap. Here, we investigated the populations in Southeastern China and eastern North America and evaluated the phylogeographical patterns from their geographical range sizes, geographic–genetic distance relationships and their responses to spatial‐environmental factors. Using the mitochondrial cytochrome c oxidase subunit I gene as the DNA marker, we analyzed a total of 170 individuals. Our results showed that some putative cryptic species, also known as entities were widely distributed, but most of them were limited to single areas. The divergence of *P*. *dolichoptera* and *P*. *vulgaris* indicated that gene flow between continents was limited while that within each continent was stronger. Oceanographic barriers do affect the phylogeographic pattern of rotifers in continental waters and serve to maintain genetic diversity in nature. The genetic distance of *P*. *dolichoptera* and *P*. *vulgaris* populations showed significant positive correlation with geographic distance. This might be due to the combined effects of habitat heterogeneity, long‐distance colonization, and oceanographic barriers. Furthermore, at the intercontinental scale, spatial distance had a stronger influence than environmental variables on the genetic differentiations of both populations. Wind‐ and animal‐mediated transport and even historical events of continental plate tectonics are potential factors for phylogeography of cosmopolitan rotifers.

## Graphical abstract

Oceanographic barriers affect the phylogeographic pattern of rotifers in continental waters and maintaining genetic diversity in nature. The divergence indicates that gene flow between eastern North America and Southeastern China is limited while that within eastern North America or Southeastern China was higher. Spatial variables are key factors in affecting the genetic differentiation of rotifers when compared with environmental variables on the intercontinental scale.

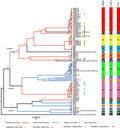

## 1 INTRODUCTION

Two of the most important questions in ecology and biogeography are how and why species composition differs between geographic locations (Dambros et al., 2020). Environmental factors, geographic distance, and dispersal barriers are potential drivers. Some researches suggest that genetic diversity is related to geographical distances by a classical distance–decay relationship (Gómez et al., 2002; Gómez‐Rodríguez et al., 2020). Others hold that niche differentiation including abiotic and biotic factors, not dispersal, controls the patterns of genetic differentiation (Jin et al., 2020; Li et al., 2020; Ma et al., 2019). But most studies have demonstrated that combining ecological and phylogeographic information is more effective in understanding the factors that drive the distribution of genetic diversity (Gabaldón et al., 2017).

Cryptic species are difficult or even impossible to distinguish by their morphology. The putative cryptic species defined through phylogenetic models by clusters of sequences are called entities (Obertegger et al., 2015). Generalized mixed Yule coalescent (GMYC), automatic barcode gap discovery (ABGD), and Poisson tree processes (PTP) models are widely used approaches for cryptic entities delimitation (Fontaneto, 2014; Kordbacheh et al., 2017). Since the sequences are clustered by different models according to the genetic distance, they are called GMYC entities. (Obertegger et al., 2015). With the development of molecular tools, increasing numbers of cryptic entities have been discovered in various morphological species of rotifers (Fontaneto, 2014). Cryptic entities have been found in almost all groups of animals (Fossen et al., 2016; Tang et al., 2012), and rotifers seem to be one of the invertebrates hosting the highest potential cryptic diversity in the world (Fontaneto, 2014; Fontaneto et al., 2009). For instance, eight potential entities of *Brachionus calyciflorus* were found in eastern China (Xiang et al., 2011). Also, more than seven entities of *Euchlanis dilatata* were defined in North America (Kordbacheh et al., 2017). By the end of 2017, there were 15 entities of *B*. *plicatilis* recorded in the world (Mills et al., 2017). Rotifers are invertebrates sensitive to environmental changes. They are widely distributed all over the world and live in all kinds of water bodies (Liang et al., 2020). Thus, rotifers are advantaged for the study of phylogeography and excellent examples of cryptic entities. As the cosmopolitan and dominant species of rotifers show strong adaptability to the environmental changes, studying their spatial and temporal patterns of genetic differentiation is of great significance for understanding gene flow and adaptive evolution mechanisms in microscopic organisms (Zhang et al., 2018).

Rotifer biogeography is a complicated issue; biogeographical patterns exist but are difficult to detect and have been studied mostly at the continental scale (Fontaneto, 2019). To date, findings of the relationship between geographical distance and genetic distance are inconsistent. A study of *Brachionus calyciflorus* phylogeography in eastern China showed no significant association between geographical and genetic distances (Xiang et al., 2011). Moreover, a significant albeit weak correlation was found in *Euchlanis dilatata* phylogeography within North America (Kordbacheh et al., 2017). The relationship between geographical distance and genetic distance is far from clear in rotifers, as seen in studies of large geographic scales.

Baas‐Becking's hypothesis, known as “everything is everywhere, but the environment selects” (EisE) posits that for small species, spatial variation occurs because of ecological differentiation and not because of restricted dispersal (Fenchel & Finlay, 2006). For example, the differential distribution of cryptic entities has been related to ecological factors: temperature and algal food concentration in the *B*. *calyciflorus* population (Li et al., 2010); salinity in the *B*. *plicatilis* population (Gómez et al., 2002); and total phosphorus concentration in the *Synchaeta pectinata* population (Obertegger et al., 2012).

Moreover, coexistence of cryptic entities is common in a single water body and even can be found in two distanlty separated locations (Fontaneto et al., 2008; Papakostas et al., 2016; Wen et al., 2016). However, most studies of environmental‐spatial selection to date focused on small geographic or continental scales, and genetic differentiation across intercontinental scales has been underexplored. For instance, the two cryptic entities belonging to the *Ascomorpha ovalis* population occur at different altitudes in Mexico area (García‐Morales & Elías‐Gutiérrez, 2013). In another example, cryptic entities of a *Polyarthra dolichoptera* population was distributed along an altitudinal gradient in Trentino–South Tyrol, Italy. Although several studies have supported the idea that most cryptic taxa within the population are widespread while others have locally restricted distributions (Fontaneto, Barraclough, et al., 2008; Mills et al., 2017), the knowledge about effects of geographical barriers on genetic differentiation of rotifer is still limited.

*Polyarthra dolichoptera* and *P*. *vulgaris*, cosmopolitan rotifer species, are more tolerant to seasonal changes than other rotifers and exist in a variety of freshwater bodies but not marine systems (Liang et al., 2019). They are zooplankton with limited mobility and can be transported by currents within a single water body. As they are monogonont rotifers that reproduce by cyclical parthenogenesis, resting eggs (dormant stages) will be produced to escape unsuitable environmental conditions to temporarily avoid competition and parasitism and thus to move to distant areas and new habitats through abiotic and biotic factors (Fontaneto, 2019). It has been suggested that *Polyarthra* entities distribution reflects genotypic adaptations to temperature differences and food resources (Obertegger et al., 2015). Higher genetic diversity is seen in low‐altitude lakes that are characterized by warm temperature and mesotrophic to eutrophic conditions (Obertegger et al., 2015). However, the major factors of genetic differentiation of rotifers at larger geographic scales is still unclear and needs further investigation.

In this study, we attempted to address whether oceanographic barriers affect the phylogeographic pattern and maintain biological diversity of rotifers in continental waters by investigating the genetic differentiation of *P*. *dolichoptera* and *P*. *vulgaris* populations from Southeastern China and eastern North America. The specific objectives were as follows: (1) to determine whether the populations of cosmopolitan species from different continents formed independent or overlapping strains; and (2) to understand whether environmental or spatial variables are the key factors affecting genetic differentiation.

## 2 MATERIALS AND METHODS

### 2.1 Sampling

**FIGURE 1:**
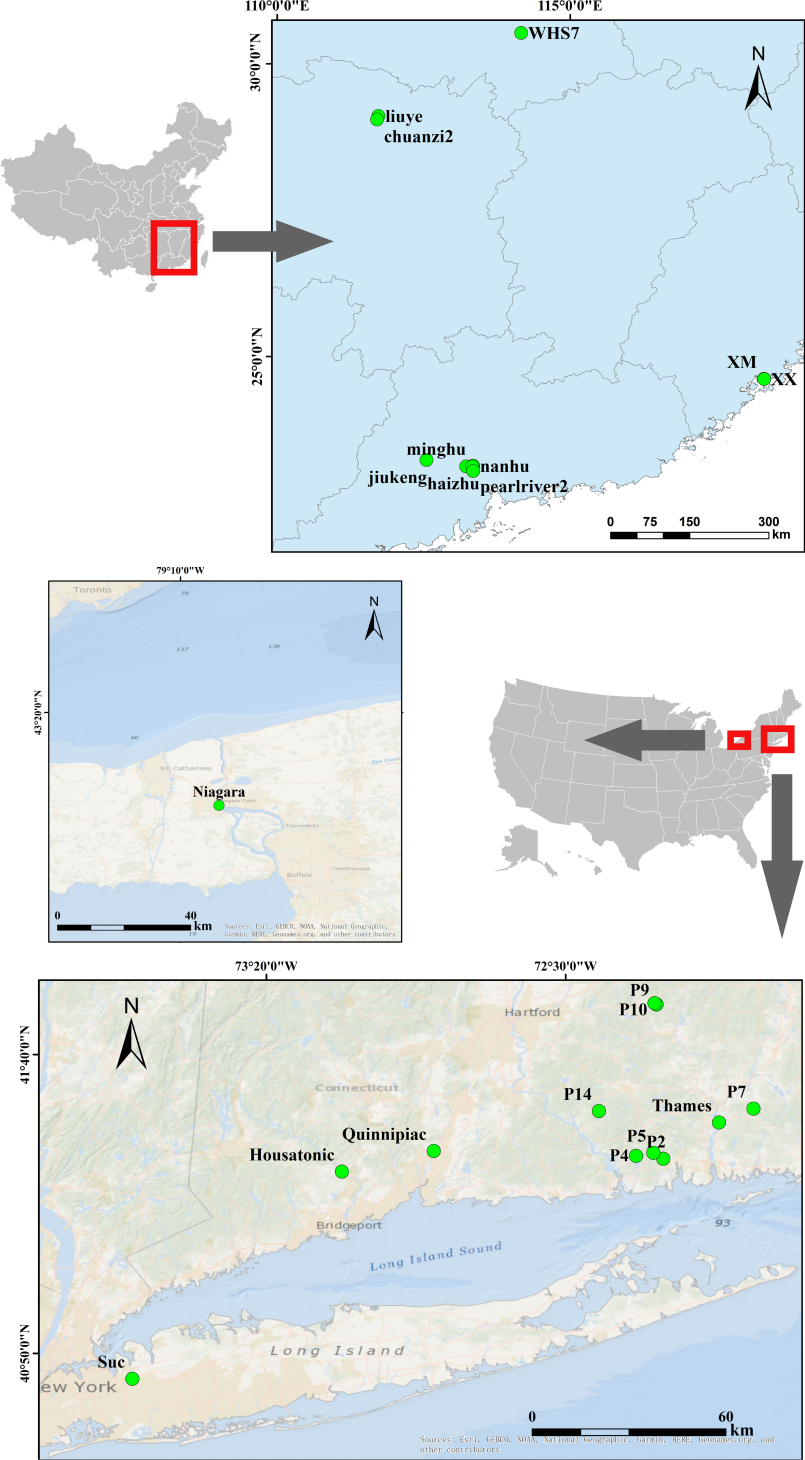
Location of the sampling sites in Southeastern China and eastern North America

Samples were collected from 28 sites including rivers, ponds, and lakes in both eastern North America and Southeastern China, during June 2018 to September 2019 (Figure 1; Table 1). For determining detailed cryptic entities structure at a small geographic scale, five sites in Guangzhou City, China, and five ponds in New London Country, USA, were sampled. In consideration of temperature effects on entities structure, four seasons were sampled in Lake Liuye, China (6_liuye, 9_liuye, 12_liuye, and 3_liuye; Table 1).

**TABLE 1:** Details of sampling localities and the environmental parameters

Sampling site	Sample name	Collection date	Individuals	Longitude	Latitude	Altitude(m)	Temperature(℃)	Chlorophyll‐a (ug/l)	TN(mg/l)	TP(mg/l)
March_Lake Liuye	3_liuye	2019.3	7	111.7291667	29.10972222	30	14	10	3.79	0.14
June_Lake Liuye	6_liuye	2019.6	21	111.7272222	29.04833333	30	28	18	10.31	0.13
September_Lake Liuye	9_liuye	2018.9	6	111.7091111	29.12	30	25	5	11.1	0.06
December_Lake Liuye	12_liuye	2018.12	1	111.7602778	29.06916667	30	8	1	3.37	0.01
September_the Chuanzi River	9_chuanzi2	2018.9	14	111.6919444	29.05388889	29	30	17	11.2	0.06
December_the Chuanzi River	12_chuanzi2	2018.12	2	111.6919444	29.05388889	29	8	1	4.5	0.07
the pond of Haizhu Park	haizhu	2018.12	9	113.2283333	23.12277778	2	11	8	3.5	0.1
the pond of Minghu	minghu	2019.1	8	113.343229	23.134201	11	17	72	1.2	0.18
the pond of Nanhu	nanhu	2019.1	3	113.344337	23.131315	15	18	18	2.3	0.15
Guangzhou segment of the Pearl River	pearlriver2	2019.3	4	113.347176	23.042379	1	17	1	0.76	0.07
the pond of Zhujaing Park	Zhujiangpark	2019.4	7	113.3338528	23.12243056	3	25	17	3.9	0.09
the reservoir of Jiukeng	jiukeng	2019.4	3	112.548204	23.231587	39	25	1	2.5	0.06
the pond of Xiamen University	XM	2018.8	11	118.3099722	24.62083333	47	27	78	4.5	0.1
the brook of Xiamen University	XX	2018.8	2	118.309304	24.613373	21	27	8	3.7	0.11
the pond near Lake Donghu	WHS7	2018.7	14	114.165	30.52861111	18	27	107	2.5	0.27
Thames river	Thames	2019.5.23	4	−72.073133	41.47805	0	16	1	1.5	0.1
Housatonic river	Housatonic	2019.9.28	3	−73.123711	41.340767	8	25	78	2.8	0.13
Quinnipiac river	Quinnipiac	2019.9.28	2	−72.867685	41.398428	−2	24	10	2.5	0.1
Lake Success	SUC	2019.6.2	7	−73.707286	40.763248	59	22	65	3.5	0.11
Niagara waterfall	Niagara	2019.8.15	3	−79.062749	43.081979	166	25	1	0.2	0.03
Pattagansett Lake	P2	2019.6.15	4	−72.228367	41.376893	20	23	8	0.3	0.1
Norwich Pond	P4	2019.6.15	2	−72.303911	41.384799	25	24	2	1.1	0.08
Powers Lake	P5	2019.6.15	2	−72.255902	41.393302	48	24	3	5.4	0.09
Amos Lake	P7	2019.7.8	3	−71.977407	41.516628	39	25	6	6.3	0.11
Mirror Lake	P9	2019.9.13	4	−72.247236	41.806832	179	24	3	3.4	0.07
Swan Lake	P10	2019.9.13	6	−72.25277	41.81083	184	24	5	5.6	0.07
Moodus Reservoir	P14	2019.9.28	3	−72.40739	41.509835	109	24	10	3.8	0.12

All rotifer samples were collected by towing a plankton net (mesh size 30 μm) horizontally at surface and subsurface depths and preserved in 50‐ml centrifuge tubes. Samples were fixed on site immediately with neutral Lugol's solution at 2% final concentration and transported in a cooler before storing at −20°C. In vivo semi‐quantitative measurements of chlorophyll‐*a* concentration (Chl‐*a*) were obtained using a FluoroSenseTM handheld fluorometer (Turner Designs, USA). Water temperature (Temp) was measured on site. Total dissolved phosphorus (TP) and total dissolved nitrogen (TN) were determined in the laboratory following standard analytical methods (GB3838‐2002, MEE, 2002). Also, GPS coordinates and altitude values were recorded.

### 2.2 Species identification and isolation

**FIGURE 2:**
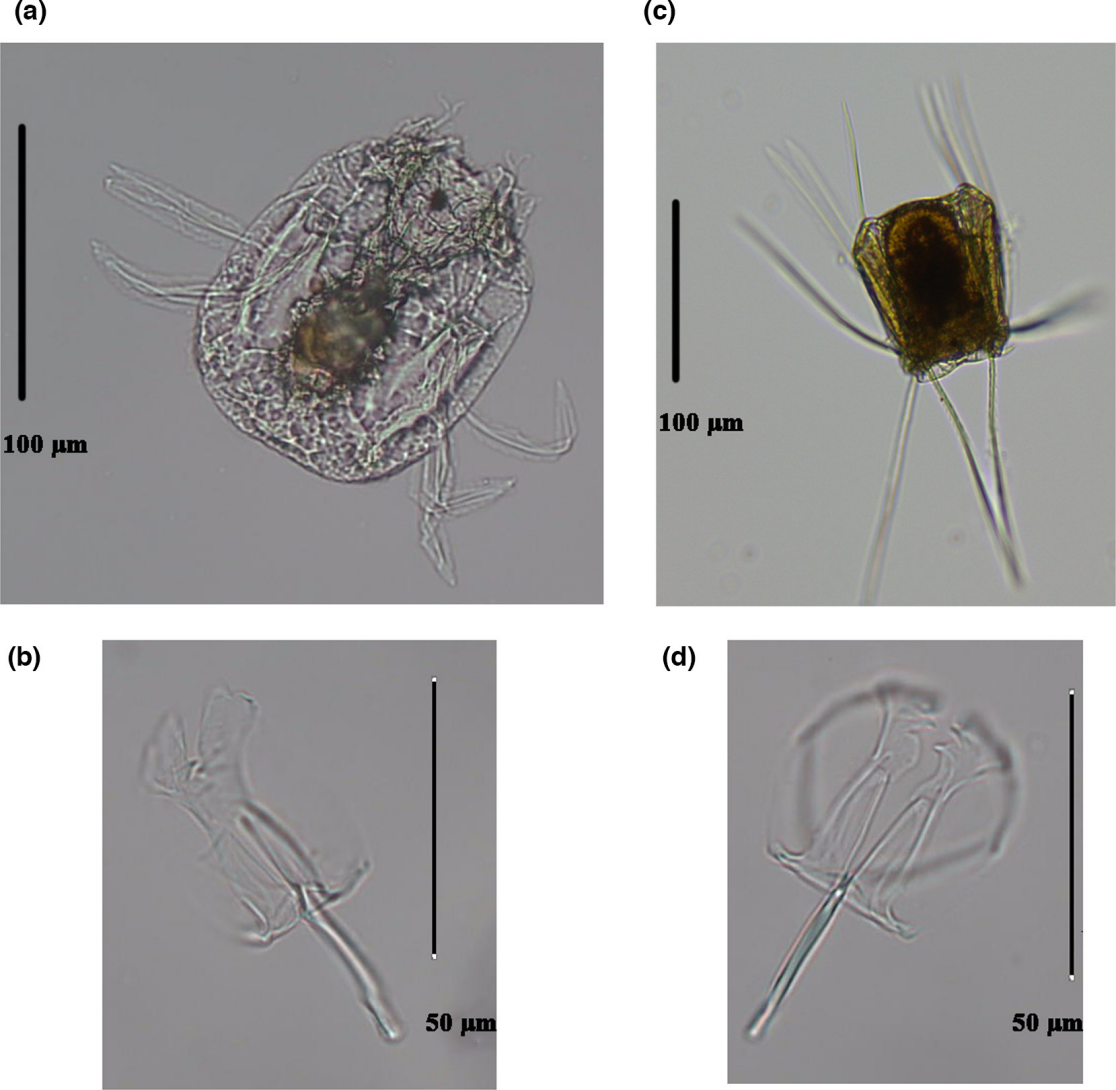
Morphologic photographs: *P*. *vulgaris* (a); the trophi of *P*. *vulgaris* (b); *P*. *dolichoptera* (c); the trophi of *P*. *dolichoptera* (d)

Species identification was based on the most authoritative taxonomy review on Synchaetidae (Nogrady & Segers, 2002) using a regular compound microscope (Olympus BX51). Although *P*. *dolichoptera* and *P*. *vulgaris* are similar in morphology, they can still be distinguished by morphological features such as body sizes and fins. For *P*. *dolichoptera*, the main fins are as long or longer than body length; width/length 1:10–1:15, serration coarse, widely spaced, uniformly sword‐like with central rib almost reaching the end; serration lines faint or absent. For *P*. *vulgaris*, the main fins are as long or shorter than body length but broader than that of *P*. *dolichoptera*; width/length 1:5–1:7, feathery or leaf‐like with strong central rib. Each identified individual was isolated with micropipette under the stereomicroscope (Olympus SZX16) (Figure 2). Single individuals were rinsed several times and transferred into PCR tubes for DNA analysis.

### 2.3 DNA extraction and amplification

DNA from each single animal was extracted following the HotSHOT protocol (Montero‐Pau et al., 2008). Then, the partial mitochondrial cytochrome c oxidase subunit I (COI) mtDNA gene was amplified and sequenced using primers LCOI (5'‐GGT CAA CAA ATC ATA AAG ATA TTGG‐3') and HCOI (5'‐TAA ACT TCA GGG TGA CCA AAA AAT CA‐3') (Folmer et al., 1994). PCR was processed according to the TaKaRa exTaq protocol with 5 μl of extracted DNA. Cycle conditions were initial denaturation at 94°C for 3 min, followed by 35 cycles of denaturation at 94°C for 30 s, annealing at 52°C for 30 s, and extension at 72°C for 45 s. The amplification ended with a final extension of 72°C for 8 min. Successful amplification products were then purified using the TaKaRa Minibest agarose Gel DNA extraction Kit before being sent to TsingKe company for sequencing.

### 2.4 Sequence alignment and phylogenetic analyses

Sequences were aligned in Mega X using Clustal‐W and then checked manually (Kumar et al., 2018). The same program was used to translate COI gene sequences to proteins and to check for stop codons. Each sequence was verified by BLAST search in NCBI GenBank (Altschul et al., 1990). Within‐species genetic distances should be <14% for mt COI (Mills et al., 2017; Obertegger et al., 2015). The closest sequences with the highest similarity scores were obtained from GenBank for comparison (Accession #: KJ460388, LC215566, LC215573, KC618934, KC619030, JN936500, KJ460383, KC619195, and LC215562). Population genetic statistics within species (average number of nucleotide differences between haplotypes, number of haplotypes, haplotype diversity [*Hd*], and nucleotide diversity [*π*], average number of nucleotide differences [K], average number of segregating sites [S]) were calculated using DNASP 5.1 (Librado & Rozas, 2009). The uncorrected (“p”) genetic distance matrix was calculated in Mega X after model testing by Modeltest 3.7 (Kordbacheh et al., 2017).

Bayesian phylogenetic trees were run in BEAST v1.8.4 (Drummond et al., 2012), separately for the two data sets (*P*. *dolichoptera* and *P*. *vulgaris* sequences). The selected model of evolution for phylogenetic reconstructions was HKY + I + G, chosen by approximate likelihood ratio tests in ModelGenerator v0.85 (Keane et al., 2006). For this analysis, an uncorrelated lognormal relaxed clock, the Yule process speciation prior (rate of linear birth in the Yule model of speciation set as lognormal) with the default settings of prior and the MCMC of 10^7^ generations with sampling every 1000 generations were used. Tracer v1.6 was used for evaluating effective sample size (ESS > 200) (Rambaut et al., 2013). Trees were summarized using TreeAnnotator v1.8.4 with a 20% burn‐in (Drummond et al., 2012). For *P*. *dolichoptera* phylogenetic reconstructions, congener *P*. *vulgaris* (KJ460388) was included as outgroup, and *P*. *dolichoptera* (KC618934) was included as outgroup for *P*. *vulgaris*.

### 2.5 Cryptic entities delimitation

An ultrametric tree generated by BEAST was required for both GMYC and PTP delimitations. The GMYC delimitation analysis was processed on software R 3.6.1 using the “rncl” and “splits” packages, (R Core Team, 2019). The GMYC model is a likelihood method for delimiting species by fitting within and between species branching models to reconstruct gene trees (Fujisawa & Barraclough, 2013; Pons et al., 2006).

The ABGD model was performed for primary species delimitation and was processed on the website https://bioinfo.mnhn.fr/abi/public/abgd/ (Accessed January 10, 2020). ABGD classifies sequences into putative entities based on pairwise genetic distances without any prior assumptions (Puillandre et al., 2012).

PTP is a tree‐based method that uses the number of substitutions to distinguish intraspecies processes from interspecies processes. This method considers two classes of Poisson processes, speciation (higher substitution rate associated to interspecies events), and coalescence (within species events) (Zhang et al., 2013). PTP (http://species.h‐its.org/ptp/, Accessed January 8, 2020) was applied to ultrametric BEAST trees using the default settings to detect the number of entities (Kordbacheh et al., 2017).

### 2.6 Geographical and genetic distance analysis

The geographic distance matrices were calculated in R 3.6.1 (R Core Team, 2019) using the “geosphere” package (Hijmans et al., 2019). In order to explain the relationships between geographic distances and genetic distances, linear regressions were processed in R 3.6.1. version 1.2.3 of the Geographic Distance Matrix Generator was used to construct a geographic distance matrix. Mantel tests were run in the R package “ecodist” with 10,000 permutations to test whether genetic variation among populations is correlated to geographic distances among populations (Kordbacheh et al., 2017). To determine the significance of differences (*p* < .05) in genetic distances among three different groups, analysis of variance (ANOVA) with TukeyHSD test was conducted, using the software R 3.6.1, “agricolae,” “car,” and “multcomp” packages.

### 2.7 Relationships between entities and environmental factors

Since the approximation by the linear function is poor over a long gradient when the data are too heterogeneous, detrended correspondence analysis (DCA) should be carried out. CCA or RDA model is determined based on the 27 sample datasets (Table 1) by detrended correspondence analysis (DCA). Individuals from each location and each season in the same location were considered as an independent dataset for analysis. After DCA analysis, redundancy analysis (RDA) or canonical correlation analysis (CCA) was performed to explore the relationships among entities (*P*. *dolichoptera* and *P*. *vulgaris*), environmental and spatial factors using the “vegan” and “ggplot2” packages in R. If the longest gradient is >4, the unimodal method (CCA) can be applied. On the other hand, if that value is <3, the linear method (RDA) is a better choice. In the range between 3 and 4, both methods can be applied (Ter Braak & Smilauer, 2002). Varying inflation factors less than 20 (VIF < 20) were included in the analysis and the envfit (permu = 999) function was used to determine the significant (*p* > .05) variables (Oksanen et al., 2010).

## 3 RESULTS

### 3.1 Genetic diversity

We obtained 107 COI sequences of *P*. *dolichoptera* and 63 sequences of *P*. *vulgaris*, with aligned lengths of 562p and 589 bp, respectively (Accession numbers: Table S1). A total of 64 *P*. *dolichoptera* haplotypes were detected with haplotype diversity (h) of 0.98 and nucleotide diversity (π) of 0.175. For *P*. *vulgaris*, a total of 36 haplotypes were found with a haplotype diversity of 0.96 and nucleotide diversity of 0.106 (Table 2).

**TABLE 2:** Genetic diversity summary statistics, as calculated by DNASP 5

Samples	Individuals	Haplotypes	Haplotype diversity (*Hd*)	Nucleotide diversity (*π*)	Average number of nucleotide differences (K)	Average number of segregating sites (S)
Total *P*. *dolichoptera*	103	64	0.979	0.175	94.87	330
*P*. *dolichoptera* in China	70	41	0.96	0.149	80.97	230
*P*. *dolichoptera* in the USA	33	23	0.978	0.156	84.51	281
Total *P*. *vulgaris*	63	36	0.957	0.106	60.73	227
*P*. *vulgaris* in China	42	24	0.93	0.048	27.56	156
*P*. *vulgaris* in the USA	21	12	0.895	0.026	15.11	91

For *P*. *dolichoptera* population, greater genetic variation was observed in eastern North America than in southeastern China, with higher haplotype diversity (*H_d_*; 0.978), nucleotide diversity (*π*; 0.156), average number of nucleotide differences (K; 84.51), and average number of segregating sites (S; 281). For *P*. *vulgaris* population, higher levels of *H_d_* (0.93), *π* (0.048), K (27.56), and S (156) were detected in the Southeastern China samples (Table 2).

### 3.2 Phylogenetic analysis and entities delimitation

**FIGURE 3:**
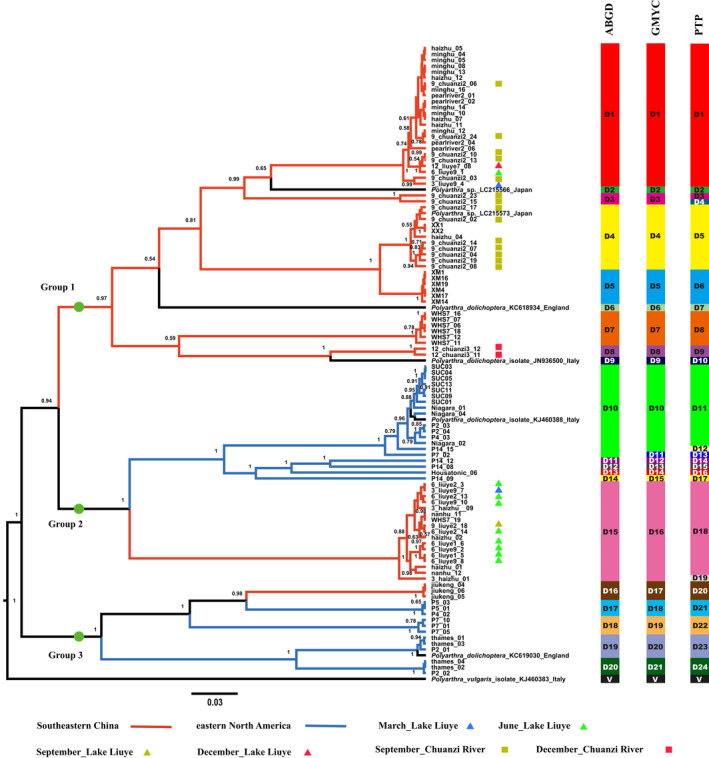
The Bayesian phylogenetic tree of the *P*. *dolichoptera* population based on 108 COI gene sequences. Posterior probabilities (>0.5) from Bayesian reconstruction are shown at each node. Putative entities detected using automatic barcoding gap discovery (ABGD), generalized mixed Yule coalescent models (GMYC), and Poisson tree process (PTP) are shown. Abbreviation: D, *P*. *dolichoptera* entities; V, *P*. *vulgaris* entities, which was included as the outgroup. The scale bar denotes three substitutions per 100 sites

**FIGURE 4:**
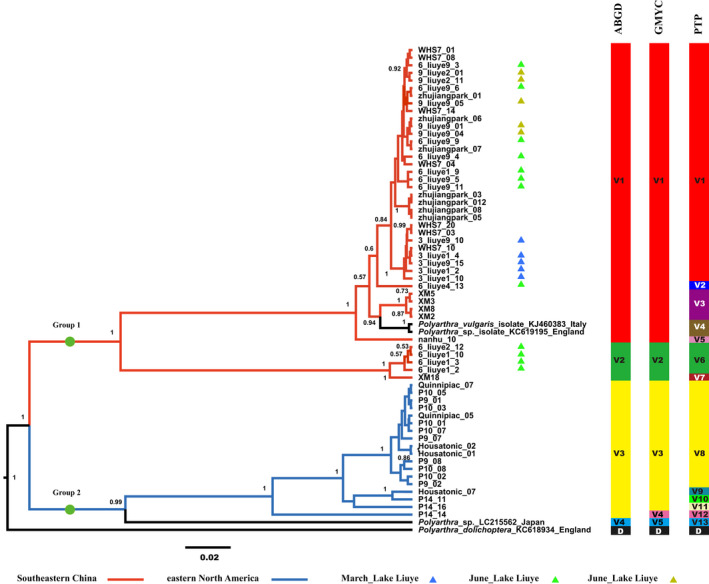
The Bayesian phylogenetic tree of the *P*. *vulgaris* population based on 64 COI gene sequences. Posterior probabilities (>0.5) from Bayesian reconstruction are shown at each node. Putative entities detected using automatic barcoding gap discovery (ABGD), generalized mixed Yule coalescent models (GMYC), and Poisson tree process (PTP) are shown. Abbreviation: V, *P*. *vulgaris* entities; D, *P*. *dolichoptera* entities, which was included as the outgroup. The scale bar denotes two substitutions per 100 sites

Using Bayesian phylogenetic analysis, *P*. *dolichoptera* population was reconstructed (Figure 3). All the individuals analyzed were binned into three groups. Group 1 (posterior probability = 0.97) was composed of 54 individuals, which were all from Southeastern China. This group was composed of similar clusters from different sites in Southeastern China. Group 2 (posterior probability = 1) consisted of 20 individuals from eastern North America and 17 individuals from Southeastern China, which formed independent clades by continents, showing high genetic divergence between the two geographic communities. Group 3 (posterior probability = 1) consisted of 13 individuals from eastern North America and three individuals from Southeastern China, which formed many divergent clusters within eastern North America and an independent clade for the individuals from Southeastern China. The Bayesian tree analysis clustered the *P*. *vulgaris* samples into two groups with strong support values (Figure 4). All of the 42 individuals from Southeastern China were in Group 1 (posterior probability = 1), while the 18 individuals from eastern North America were in Group 2 (posterior probability = 0.99) with strong support values.

A large number of cryptic entities were detected using three independent methods (Figures 3 and 4). The ABGD method produced 20 cryptic entities for *P*. *dolichoptera* population and four for *P*. *vulgaris*. GMYC analysis revealed 21 cryptic entities in *P*. *dolichoptera* population and five in *P*. *vulgaris* population. Using the PTP method, *P*. *dolichoptera* population was delimited into 24 entities and *P*. *vulgaris* population into 13. The most conservative estimate of cryptic entities was obtained using ABGD, while the PTP method gave the greatest number of cryptic entities. All three methods shared common species boundaries for the smallest number of cryptic entities, however. As the results from ABGD and GMYC were similar, unless specified otherwise, cryptic entities determination will be discussed based on ABGD results (ABGD entities).

### 3.3 Geographical distribution and coexistence

The range size of geographic distances declined as the resolution of classification increased from morphological species to cryptic entities to haplotype (Figure S1). Both *P*. *dolichoptera* and *P*. *vulgaris* species were widely distributed, with the geographic range sizes up to 12,903 and 12,836 km, respectively. Their range sizes at cryptic entities and haplotype levels decreased to 2726 and 411 km, respectively. These correspond to the mean range sizes at species level (*P*. *dolichoptera*: 5374 ± 5665 km; *P*. *vulgaris*: 5517 ± 5720 km) significantly larger than at entities (463 ± 815 km) and haplotype level (12 ± 61 km) (*p* < .05). However, there was no significant difference in range size between entities and haplotype ranges.

Most of the entities were limited to single areas, but some were widely distributed. For example, entity one of *P*. *vulgaris* population (V1) comprised individuals from as distant areas as Changde (liuye), Wuhan (WHS7), Xiamen (XM), and Guangzhou (zhujiangpark), in China (Figure 4). In addition, entity 10 of *P*. *dolichoptera* population (D10) was as widely distributed as from Connecticut (P4), Long Island (SUC), and Niagara (upstate New York), in United States. In contrast, some entities only occurred in one sampling site, such as D7 and D16 (Figure 3). These results indicated that the entities and haplotypes tended to be regionally restricted. Although some of them can be widely spread into different habitats (sampling sites), no entity or haplotype was found to occur on both continents.

Coexistence and seasonal succession also occurred in the present study. D1 and D15 coexisted in June in Liuye Lake, while D1, D3, and D4 coexisted in September in the Chuanzi River (Figure 3). V1 and V2 also coexisted in June in Liuye Lake (Figure 4). On the other hand, D8 only appeared in December in the Chuanzihe River.

### 3.4 Phylogeographical patterns and genetic structure

**FIGURE 5:**
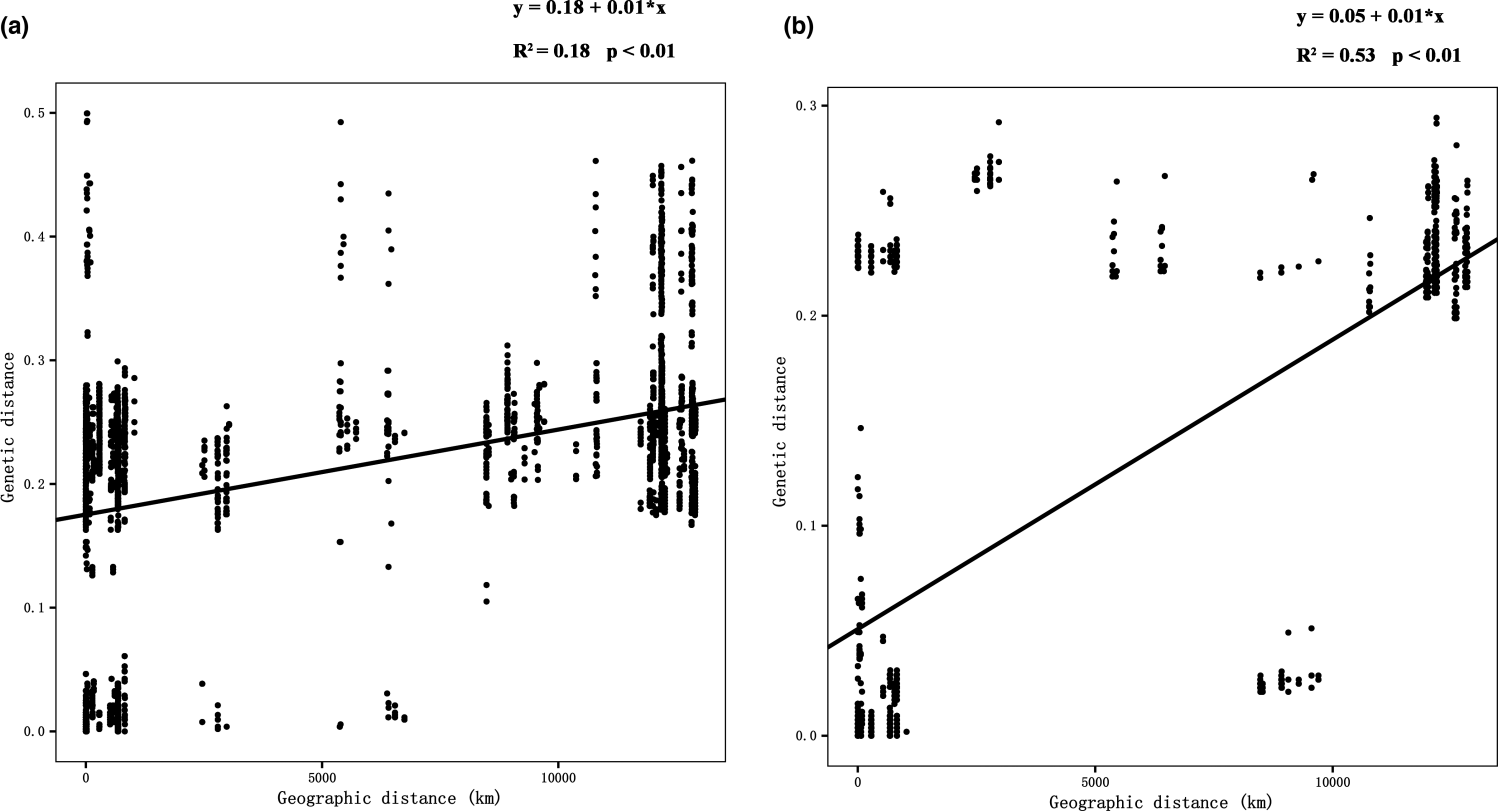
Linear regression model between the uncorrected pairwise genetic distances and geographic distance of the *P*. *dolichoptera* populations (a) (*n* = 5724) and the *P*. *vulgaris* populations (b) (*n* = 1984)

The relationships between dependent variables for genetic distance and independent variables for geographic distance were examined by linear regression. The genetic distance of *P*. *dolichoptera* population showed significant positive correlation with geographic distance (*R* ^2^ = .18, *p* < .01) (Figure 5a). The genetic distance of *P*. *vulgaris* population was also positively correlative with geographic distance (*R* ^2^ = .53, *p* < .01) (Figure 5b).

**FIGURE 6:**
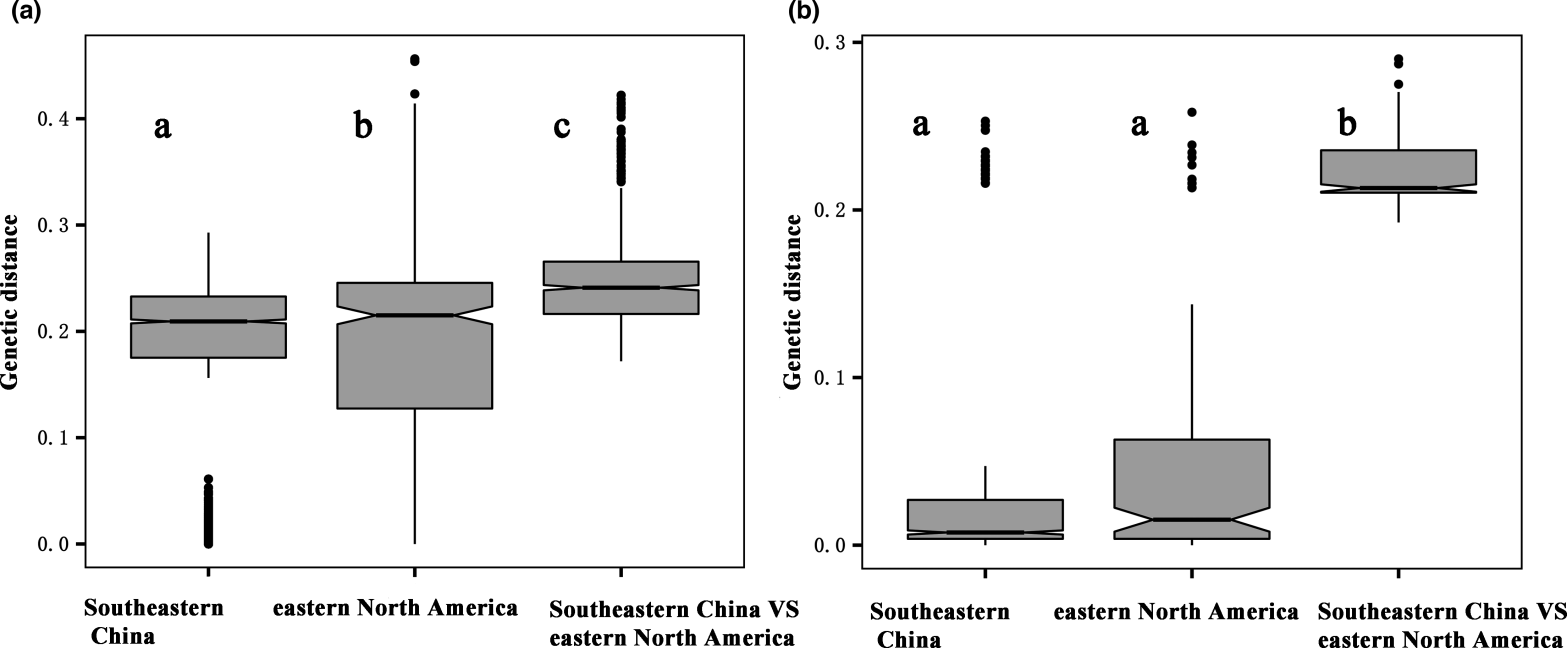
The ranges of uncorrected pairwise genetic distances among the three groups of *P*. *dolichoptera* (a) and *P*. *vulgaris* (b). Differences were detected with the Turkey HSD method. Letters indicate sample means that are similar (same letter) or significantly different (different letter)

Our results indicated that in both *P*. *dolichoptera* and *P*. *vulgaris* populations, the mean genetic distances between the two continents were significantly higher than those within either eastern North America or Southeastern China (*p* < .05) (Figure 6). The mean genetic distance in the *P*. *dolichoptera* population decreased in the order: Southeastern China VS eastern North America (0.248 ± 0.052) > within eastern North America (0.188 ± 0.111) > within Southeastern China (0.173 ± 0.089) (Figure 6a). The mean genetic distances in the *P*. *vulgaris* population decreased in the order: Southeastern China VS eastern North America (0.223 ± 0.02) > within Southeastern China (0.055 ± 0.088) > within eastern North America (0.049 ± 0.067) (Figure 6b). However, there was no significant difference in the mean genetic distance values for the Southeastern China and the eastern North America groups in the *P*. *vulgaris* population. These results indicated that the genetic divergences between these two continents were significantly higher than those within a single continent (*p* < .05). This suggests that there is a higher level of gene flow and higher frequency of recombination within continents than between continents.

### 3.5 Relationships among entities distributions, spatial and environmental factors

**FIGURE 7:**
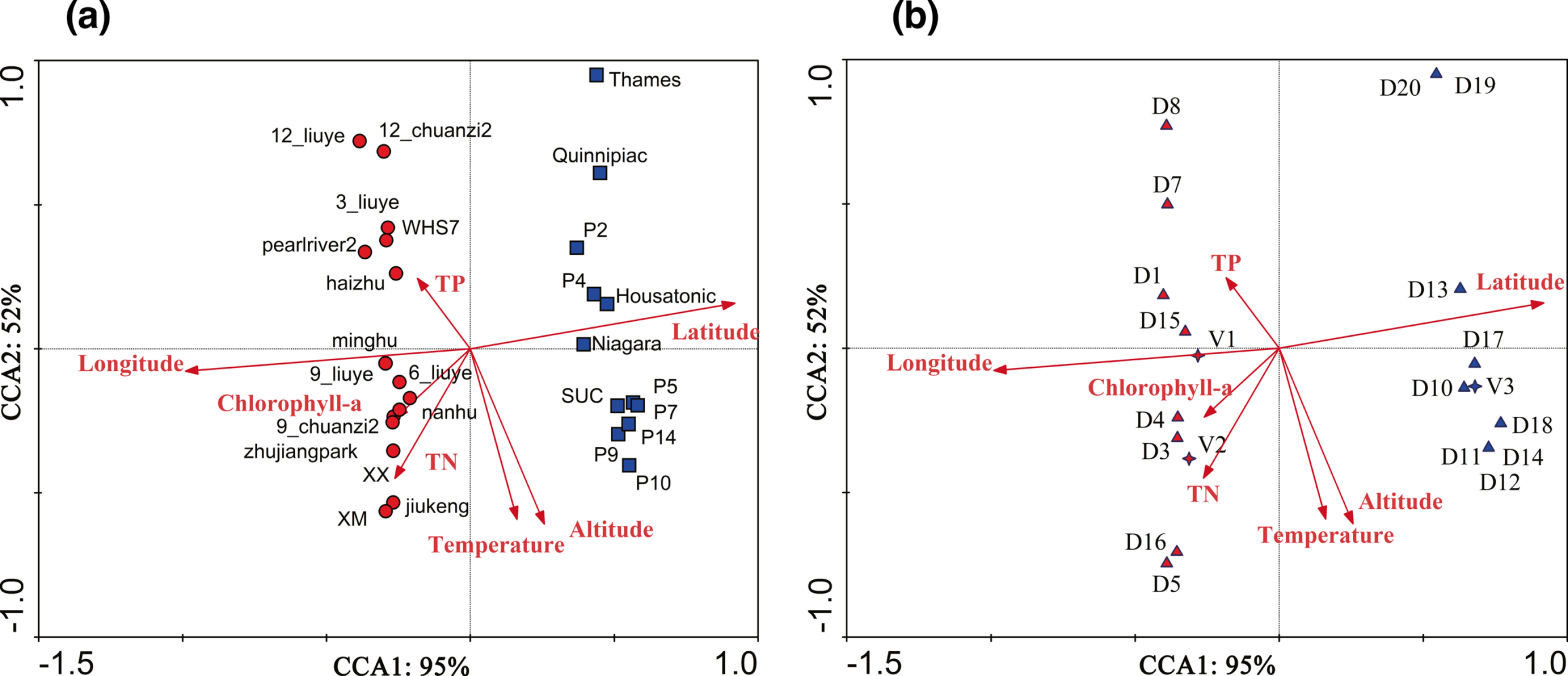
Canonical correlation analysis (CCA) of the *P*. *dolichoptera and P*. *vulgaris* entities with environmental and spatial variables. Relationship between sampling sites and variables (a). Relationship between entities and variables (b). Red, samples and entities from Southeastern China; Blue, samples and entities from eastern North America. Abbreviations used in the figures: V, *P*. *vulgaris* entities; D, *P*. *dolichoptera* entities; 3_liuye, in Lake Liuye in March; 6_liuye, in Lake Liuye in June; 9_liuye, in Lake Liuye in September; 12_liuye, in Lake Liuye in December; 9_chuanzi2, in the Chuanzi River in September; 12_chuanzi2, in the Chuanzi River in December

As the longest gradient performed by detrended correspondence analysis (DCA) was 7.5 (larger than 4, Table S2), a canonical correlation analysis (CCA) model was chosen for estimating the relationship among entities (within the *P*. *dolichoptera* and *P*. *vulgaris*), spatial and environmental factors. The first two ordinate axes explained 49.5% of the entities distribution, spatial and environment variability in the CCA ordination (Table S3). The correlation coefficient of CCA ordination showed that three variables including longitude, latitude, and altitude were significantly related to the entities distribution (*p* < .05) (Table S4). However, the environmental factors that include temperature, chlorophyll‐*a*, TN, and TP concentration were not significant variables affecting the entities’ distributions (Table S4). Figure 7 clearly showed that the Southeastern China samples (red) mostly stayed on the left of the figure, while the eastern North America samples (blue) were mostly on the right of the figure. In addition, the spatial variables of longitude were the longest and showed positive correlation with axis 1 (with the 95% eigenvalues), which indicated that longitude was the key factor for the variation of the entities’ distributions for both species. Furthermore, the entities–variables relationship was similar to that of sampling sites‐variables, which indicated that most entities (within the *P*. *dolichoptera* and *P*. *vulgaris*) tended to be restricted to specific regions.

## 4 DISCUSSION

### 4.1 Selection of DNA markers for genetic differentiation studies

Nuclear (18S, 28S) ribosomal RNA genes, nuclear internal transcribed spacer (ITS), and mitochondrial cytochrome c oxidase subunit I gene (COI) are widely used as DNA barcodes for identification (Papakostas et al., 2016). COI has been used as the most popular marker of genetic diversity in animals and was elected as the standardized tool for molecular taxonomy and identification over the last three decades (Ratnasingham & Hebert, 2007). In addition, COI showed great applicability in the study of zooplankton gene flow and thermal adaptation (Sasaki & Dam, 2019). Our results also showed that COI performed well in genetic diversity studies of *P*. *dolichoptera* and *P*. *vulgaris*.

Using one single marker, especially COI usually causes some problems due to its vulnerability to the selection process. The lack of recombination for this marker makes the data sensitive to selective sweeps. This is not ideal if one wishes to investigate the demographic processes in a population (Galtier et al., 2009). Also, the use of 18S rDNA for species in rotifers is discouraged, because of its low variability (Tang et al., 2012). ITS has been suggested to be a more reliable marker for cryptic species delimitation of *Brachionus* (Mills et al., 2017; Papakostas et al., 2016). But given that COI is more variable than ITS, the former is still the best marker to be used for exploration of population genetic structure within species and phylogeography (Mills et al., 2017). So it is better to combine COI with internal transcribed spacer (ITS) for phylogeography and cryptic species delimitation (Kordbacheh et al., 2017).

### 4.2 Genetic divergence and geographic distribution

Long‐distance dispersal of entities has been reported not only in Monogononta including *Brachionus*, *Polyarthra*, *Euchlanis*, and *Lecane* but also in Bdelloidea including *Philodina* and *Rotaria* (Fontaneto et al., 2008; Kordbacheh et al., 2017). The entity D1 was found in Guangdong and Hunan provinces, separated by >500 km (e.g., liuye, chuanzi2, minghu, pearlriver2). Also, D10 was found across a range of >500 km in the US states of New York and Connecticut (e.g., SUC, Niagara, P2, P4). In addition, V1 was widely distributed in southeastern of China, while V3 was widespread in Connecticut.

The cosmopolitan distribution of rotifers could be attributed to long‐distance dispersal. Colonization and long‐distance dispersal to different waters across whole or even multiple continents, which may be mediated by waterfowl, have been observed in a number of zooplankton species (Gómez et al., 2002). Some areas that are widely separated share haplotypes and therefore appear genetically connected (Xiang et al., 2011). Sasaki and Dam (2019) found that the widely distributed genetic clades of a marine copepod shared haplotypes between geographically distant populations. This implies that gene flow can be strong enough to overcome long distances at least within a continent.

Most small organisms do have very widespread distributions, but some are limited to distinct geographical areas (Fontaneto, Barraclough, et al., 2008; Savary et al., 2018). Our results showed that the D5 and D7 entities of *P*. *dolichoptera* only occurred in Xiamen and Wuhan, respectively. This is consistent with the study of *Brachionus calyciflorus* cryptic diversity in eastern China. Though most entities of *Brachionus calyciflorus* are widely distributed, one clade was only found in Danzhou, China (Xiang et al., 2011). Although high genetic distances of *Adineta* can be found at different geographical distances, closely related individuals were only found at geographical scales <2000 km (Fontaneto, Barraclough, et al., 2008). In the current study, we found that the range sizes of geographic distance in both genera declined as resolution increased from species to entities to haplotype. This suggests that the restricted entities in our study are not simply an artifact of sampling fewer individuals at lower levels.

Interestingly, even though *Polyarthra* was widely distributed as a genus, no identical entities or haplotypes have been found to appear on both continents at the same time. High levels of nucleotide diversity could be caused by a particularly high mutation rate which results in genetic divergence (Xiang et al., 2011). The nucleotide diversity of the total *P*. *dolichoptera* and total *P*. *vulgaris* samples were relatively higher than those from the single continent, which suggested high genetic divergence between the samples from the different continents. Given that the small sample sizes in this study impeded a thorough detection of genetic diversity, the degree of cryptic diversity in Southeastern China and eastern North America is likely to be higher than what we reported here. This genetic diversity could explain why cosmopolitan rotifers have apparently wide tolerance toward spatial and environmental changes.

Our results indicated that all of the entities from eastern North America formed independent strains that were separate from the Chinese ones, indicating high divergence. These results are consistent with the study in *Noctiluca*, a heterotrophic dinoflagellate (Pan et al., 2016). The haplotypes of *Noctiluca* within China were geographically quite homogeneous, but were generally different, compared to the American population, suggesting basin or continental‐scale endemism. In addition, a study of the entities of *B*. *plicatilis* revealed existence of four clades associated to four geographic regions (one in North America, two in Europe and one in Australia) (Mills et al., 2017).

Levels of gene flow can be estimated by producing visible patterns using allele frequencies and DNA sequence differences (Slatkin, 1987). Lack of differentiation in mitochondrial COI sequences of geographically distant populations usually indicates strong effects of gene flow (Sasaki & Dam, 2019). Our study showed that the genetic distances of Southeastern China VS eastern North America were significantly higher than those within each continent. The divergence of populations between Southeastern China and eastern North America indicates limited gene flow between the two continents. The relatively low genetic divergence in the populations within continents of both *P*. *dolichoptera* and *P*. *vulgaris* suggests strong gene flow within Southeastern China and within eastern North America.

### 4.3 Relationship between geographic and genetic distance

In the present study, a significantly positive correlation between genetic and geographic distance was found in both *Polyarthra* species. Similar results have been obtained for *E*. *dilatata* in North America (Kordbacheh et al., 2017). Moreover, it was reported that there was a strong positive correlation between genetic distance and geographic range when comparing samples at small geographical scales (Kordbacheh et al., 2017). Habitat heterogeneity and temporal variation can generate high genetic diversity on small geographic scale study (Fontaneto et al., 2009).

However, as the geographical scope of the study becomes broader, this correlation may weaken or disappear. In another example, no significant associations between geographical and genetic distances were found for *B*. *calyciflorus* across eastern China. The nonsignificant correlation may result from the effects of long‐distance colonization and secondary contact, combined with monopolization effects which reduce gene flow among established populations (Kordbacheh et al., 2017; Xiang et al., 2011). In our results, Guangdong and Hunan provinces shared *P*. *dolichoptera* entities D1 and *P*. *vulgaris* entities V1, while New York and Connecticut states shared the D10 and V3. Thus, long‐distance intra‐continental dispersal and colonization are responsible for depressing the geographic–genetic correlation in the present study.

The studies of phylogeography of rotifers usually show a weak correlation between geographical and genetic distances within continents (Kordbacheh et al., 2017; Xiang et al., 2011). Since the between‐continent genetic distances were significantly higher than those within continent, a stronger positive correlation between genetic and geographic distance was observed, in consideration of datasets from the two continents. Furthermore, the significant genetic difference between the trans‐Pacific regions suggests gene flow limitation (Pan et al., 2016). Therefore, effects of the Pacific barrier leads to the restriction of gene flow and results in an increase in genetic distance.

The relationship between geographic distance and genetic distance may not be simply linear. The results of linear regression showed that genetic distance increases rapidly with geographical distance at small geographic scales because of the coexistence and habitat heterogeneity among lakes, streams, and rivers. As the geographic distance extends to an entire continent, long‐distance colonization leads to the decrease of the genetic distance. Thus, a power‐law model is expected when there is no dispersal limitation (Gómez‐Rodríguez et al., 2020). However, extreme barriers to dispersal, such as separate oceanographic basins, lead to an increase in genetic distance at larger geographic scales. Outliers above the correlation line between geographic distance and genetic distance might suggest a significant dispersal barrier at large geographic scales.

However, the small sample sizes in this study impeded a definitive evaluation of genetic diversity. A more complete picture of the genetic and phylogeographic patterns of the rotifers in question could be established if samples were also obtained on Africa, Oceania, and the west coast of North America.

### 4.4 Key factors for phylogeographical patterns of rotifers

CCA indicated that spatial variables including longitude, latitude, and altitude were key factors in controlling the entities’ structure rather than environmental factors such as temperature TP, TN, and chlorophyll‐*a* concentrations. For most populations, genetic differentiation depend largely upon the evolutionary force regulating spatial patterns rather than seasonal differentiation (Obertegger et al., 2015; Xiang et al., 2011). In one study, cryptic diversity of *P*. *dolichoptera* was found to change along an altitudinal gradient in the Trentino–South Tyrol region, but environmental parameters such as temperature and trophic status might also affect the distribution of entities (Obertegger et al., 2015). Thus, in the absence of geographical barriers, genetic divergence might be more explained by environmental gradients (Tisthammer et al., 2020).

The present study compared populations with similar food resource levels (Chl‐*a*) at different sites and we found that the haplotypes belonged to different clades. Therefore, food sources level might not be an influencing factor for their genetic divergence. Dispersal, genetic diversity and gene flow can be strongly affected by temperature changes (Sasaki & Dam, 2019). In our study, no winter samples were collected because lakes in eastern North America were frozen over the winter. Although the ambient temperatures of our samples ranged from 8 to 30℃ (Table 1), *Polyarthra* rotifers in eastern North America actually experienced even greater interannual changes in temperature. In the future, high‐frequency and long‐period sampling surveys can be carried out in a single water body to further explore the influencing factors of the coexistence and seasonal succession of *Polyarthra* spp.

Although freshwater rotifers are already confined to an enclosed area, gene flow can be increased in geographically distant areas within the continent through wind‐, water‐, and animal‐mediated factors. Nevertheless, a quantification of the role of such potential long‐distance dispersal on the biogeography of microscopic aquatic animals is unavailable now (Fontaneto, 2019). Rotifers possess the ability for passive long‐distance dispersal through their diapausing stages including diapausing eggs and xerosomes (Walsh et al., 2017). Even a small number of successful dormant propagules is predicted to allow for effective long‐distance dispersal, especially if those propagules can rapidly develop large populations via parthenogenesis (Fontaneto, 2019). In this way, hydrology influencing community composition and wind influencing dispersal could also play an important role in rotifer dispersal (Liang et al., 2019; Rivas et al., 2018). In addition, as boating is one of the popular recreational activities for Americans in summer, rotifers and resting eggs can spread over North American lakes by launching boats. Human‐mediated transport has likely facilitated the species’ persistence since its initial colonization, through the ongoing introduction and intercontinental spread of genetic variation (Baird et al., 2020). Wind‐ and migratory‐mediated transport could operate at larger scales but are impeded by the oceanic barriers. Since dust storms and waterfowl cannot cross the strong oceanographic barriers, gene flow via dispersal of diapausing eggs and xerosomes among rotifer populations could be limited.

The phylogeographic pattern of *Polyarhtra* spp. at a global scale may also be related to the historical events of continental plate tectonics. The biogeographic realms of rotifers can be defined into eight regions (e.g., Palaearctic, Nearctic, and Oriental) (Segers, 2007). During the Cretaceous, the North American continent was still connected to the Eurasian continent, and zooplankton such as rotifers spread widely in Laurasia (Segers, 2007; Wu, 2012). In the early Cenozoic, as the North American continent drifted away from the Eurasian continent, gene flow in the established populations between the two continents was likely impeded. Southeastern China, now part of the Oriental Region, originally belonged to Gondwana. It was closely integrated with the Eurasian continent after the collision of the Indian subcontinent. Subsequently, a large amount of long‐distance dispersal and gene flow took place between the rotifer species in the Oriental region and the Palaearctic region (Wu, 2012). Since the time scales involved in the movement of the continents via plate tectonics are extremely long, however, contemporary distribution are more likely due to recent dispersal mechanisms.

## 5 CONCLUSIONS

1. Cryptic diversities of *Polyarthra dolichoptera* and *P*. *vulgaris* are definitely underestimated in the world. Oceanographic barriers do affect the phylogeographic pattern of rotifers in continental waters and serve to maintain genetic diversity in nature.
2. Entities of *P*. *dolichoptera* and *P*. *vulgaris* from eastern North America formed independent strains that were separate from the Chinese ones, indicating high divergence. The divergence indicates that gene flow between eastern North America and Southeastern China is limited while that within eastern North America or Southeastern China was higher.
3. The genetic distance of both *P*. *dolichoptera* and *P*. *vulgaris* populations showed significant positive correlation with geographic distance at the intercontinental scale. This may result from the effects of habitat heterogeneity, long‐distance colonization, and oceanographic barriers to dispersal.
4. Spatial variables are key factors in affecting the genetic differentiation of rotifers when compared with environmental variables at the intercontinental scale. Wind‐ and animal‐mediated transport and even historical events of continental plate tectonic are potential factors for phylogeographic patterns of cosmopolitan rotifers.

## CONFLICT OF INTEREST

None declared.

## AUTHOR CONTRIBUTION

**Diwen Liang:** Formal analysis (lead); Investigation (lead); Methodology (equal); Resources (lead); Software (lead); Writing – original draft (lead). **George McManus:** Writing – review & editing (equal). **Qing Wang:** Writing – review & editing (equal). **Xian Sun:** Writing – review & editing (equal). **Zhiwei Liiu:** Software (equal). **Senjie Lin:** Supervision (equal); Writing – review & editing (lead). **Yufeng Yang:** Project administration (lead); Supervision (lead).

## Supporting information

Supplementary Material

## Data Availability

The COI sequences lacked internal stop codons of this study have been submitted to the NCBI database. The GenBank accession numbers are listed in Table S1.
