# Supplementary material for: Genetic differentiation and phylogeography of rotifer Polyarthra dolichoptera and P. vulgaris populations between Southeastern China and eastern North America: High intercontinental differences
Source: Ecol Evol. 2022 May 13;12(5):e8912. doi: 10.1002/ece3.8912 (PMC9101598; doi:10.1002/ece3.8912)
Supplement: Supplementary file 1 — Supplementary Material [file ECE3-12-e8912-s001.docx]

**Supplemental Information for:**

**Genetic differentiation and phylogeography of *Polyarthra dolichoptera* and *P. vulgaris* populations between Southeastern China and eastern North America: high intercontinental differences**

**Table of Contents**

| **Supplementary Figure S1** | Page 2 |
| --- | --- |
| **Supplementary Table S1** | Page 2 |
| **Supplementary Table S2** | Page 7 |
| **Supplementary Table S3** | Page 7 |
| **Supplementary Table S3** | Page 7 |


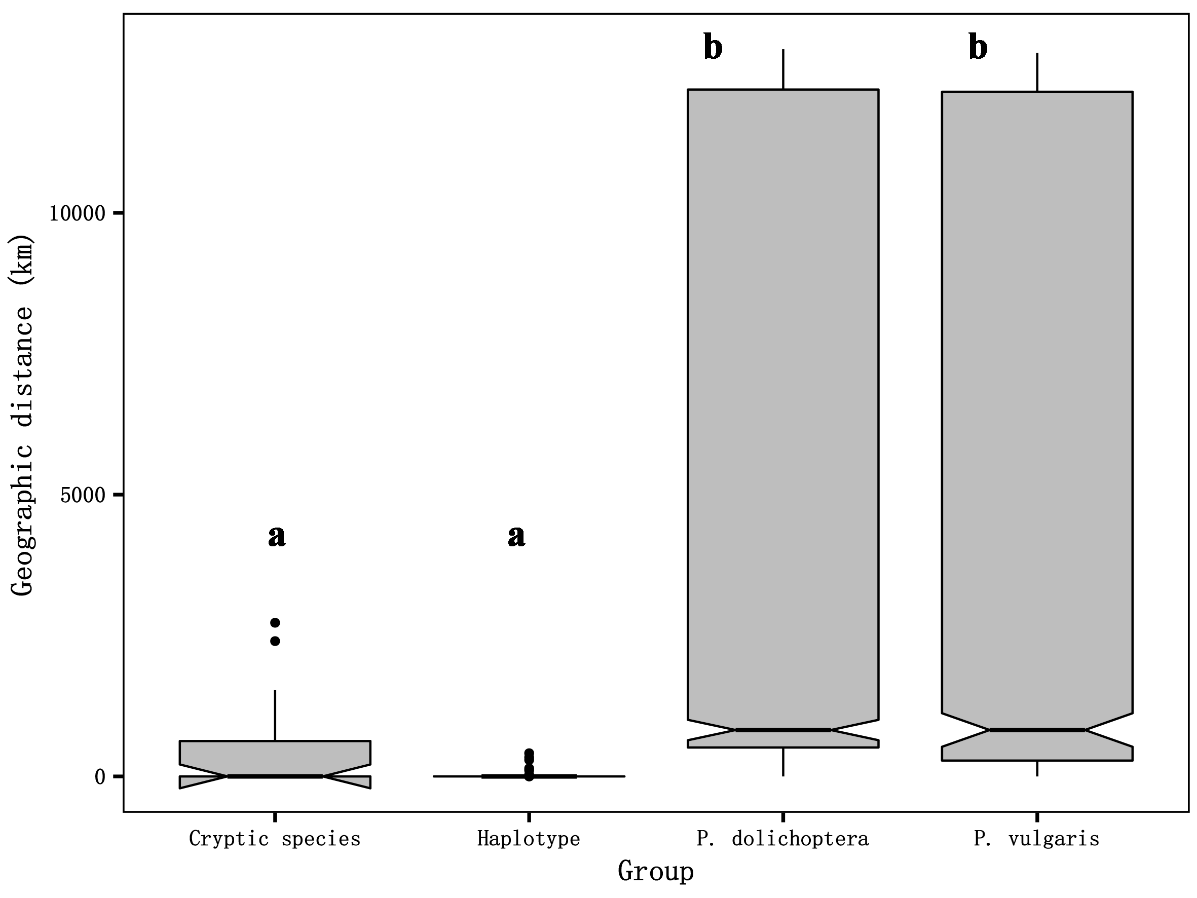


Fig. S1 Boxplot of the distribution of range size of distances in kilometers for three levels of analysis: haplotype, cryptic species and the morphological species (*P. dolichoptera* and *P. vulgaris*). Differences were detected with the TukeyHSD method. Letters indicate sample means that are similar (same letter) or significantly different (different letter).

**Supplementary Table S1** GenBank accession numbers for the corresponding COI gene sequences of the *Polyarthra dolichoptera* and *P. vulgaris* populations.

| Sampling site | Samlple name | Collection date | Longitude | Latitude | GenBank accession numbers (*P. dolichoptera*) | GenBank accession numbers (*P. vulgaris*) |
| --- | --- | --- | --- | --- | --- | --- |
| March_Lake Liuye | 3_liuye | 2019.3 | 111.72917 | 29.109722 | MT152836; MT152624; | MT313182; MT251495; MT230900; MT159574; MT136726; |
| June_Lake Liuye | 6_liuye | 2019.6 | 111.72722 | 29.048333 | MT179718; MT179716; MT179714; MT152899; MT152838; MT152837; MT152621; MT152292; MT152262; | MT337740; MT313185; MT313184; MT313183; MT251182; MT238121; MT233437; MT233436; MT233435; MT233425; MT233429; MT233428; |
| September_Lake Liuye | 9_liuye | 2018.9 | 111.70911 | 29.12 | MT181611; | MT264916; MT264917; MT264912; MT258535; MT258494; |
| December_Lake Liuye | 12_liuye | 2018.12 | 111.76028 | 29.069167 | MT191338; |  |
| September_the Chuanzi River | 9_chuanzi2 | 2018.9 | 111.69194 | 29.053889 | MT328823; MT188554; MT188553; MT188377; MT188376; MT186675; MT186643; MT186640; MT186641; MT186642; MT186639; MT181606; MT181605; MT181604; |  |
| December_the Chuanzi River | 12_chuanzi | 2018.12 | 111.69194 | 29.053889 | MT215134; MT215117 |  |
| The pond of Haizhu Park | haizhu | 2018.12 | 113.22833 | 23.122778 | MT345572; MT338284; MT338283; MT328826; MT328825; MT328824; MT249133; MT214101; MT191340; |  |
| The pond of Minghu | minghu | 2019.1 | 113.34323 | 23.134201 | MT196221; MT193009; MT193007; MT193008; MT193004; MT192941; MT192939; MT191337; |  |
| The pond of Nanhu | nanhu | 2019.1 | 113.34434 | 23.131315 | MT191339; MT191336; | MT264913; |
| Guangzhou segment of the Pearl River | pearlriver2 | 2019.3 | 113.34718 | 23.042379 | MT240312; MT214996; MT214989; MT214893; |  |
| The pond of Zhujaing Park | zhujiangpark | 2019.4 | 113.33385 | 23.122431 |  | MT269796; MT269795; MT269792; MT269794; MT264918; MT264915; MT264914; |
| The reservoir of Jiukeng | jiukeng | 2019.4 | 112.5482 | 23.231587 | MT215135; MT215136; MT215118; |  |
| The pond of Xiamen University | XM | 2018.8 | 118.30997 | 24.620833 | MT179719; MT179713; MT179717; MT179715; MT179317; MT179316; | MT251878; MT251435; MT246866; MT246785; MT246786; |
| The brook of Xiamen University | XX | 2018.8 | 118.3093 | 24.613373 | MT179712; MT179318; |  |
| The pond near Lake Donghu | WHS7 | 2018.7 | 114.165 | 30.528611 | MT181615; MT181614; MT181613; MT181612; MT181609; MT181610; MT178806; | MT258531; MT258532; MT258495; MT253596; MT253592; MT253593; MT251493; |
| Thames river | Thames | 2019.5 | -72.07313 | 41.47805 | MT280700; MT280699; MT278103; MT278102; |  |
| Housatonic river | Housatonic | 2019.9 | -73.12371 | 41.340767 |  | MT280787; MT276277; MT276276; |
| Quinnipiac river | Quinnipiac | 2019.9 | -72.86769 | 41.398428 |  | MT280789; MT280788; |
| Lake Success | SUC | 2019.6 | -73.70729 | 40.763248 | MT276284; MT276283; MT276282; MT276281; MT276280; MT276279; MT276278; |  |
| Niagara waterfall | Niagara | 2019.8 | -79.06275 | 43.081979 | MT272347; MT272346; MT272343; |  |
| Pattagansett Lake | P2 | 2019.6 | -72.22837 | 41.376893 | MT313196; MT313195; MT280701; MT278104; |  |
| Norwich Pond | P4 | 2019.6 | -72.30391 | 41.384799 | MT313197; MT309538; |  |
| Powers Lake | P5 | 2019.6 | -72.2559 | 41.393302 | MT309540; MT309539; |  |
| Amos Lake | P7 | 2019.7 | -71.97741 | 41.516628 | MT299916; MT299915; MT299914; |  |
| Mirror Lake | P9 | 2019.9 | -72.24724 | 41.806832 |  | MT272349; MT272348; MT272344; MT269791; |
| Swan Lake | P10 | 2019.9 | -72.25277 | 41.81083 |  | MT276275; MT276274; MT273664; MT273661; MT273662; MT272345; |
| Moodus Reservoir | P14 | 2019.9 | -72.40739 | 41.509835 | MT313198; | MT299926; MT299925; MT299924; |

**Supplementary Table S2** Summary statistics for the axes of DCA performed on entities

| Axes | CCA1 | CCA2 | CCA3 | CCA4 |
| --- | --- | --- | --- | --- |
| Eigenvalues | 1 | 0.9448 | 0.8951 | 0.6642 |
| Decorana values | 1 | 0.9291 | 0.8986 | 0.6760 |
| Lengths of gradient | 1 | 7.4863 | 6.5050 | 0.9028 |

**Supplementary Table S3** Summary statistics for the axes of CCA performed on cryptic species with environmental variables

| Axes | CCA1 | CCA2 | Total variance |
| --- | --- | --- | --- |
| Eigenvalues | 0.946 | 0.517 | 8.87 |
| Species-environment correlations | 0.988 | 0.848 |  |
| Cumulative percentage variance：of species data | 10.7 | 16.5 |  |
| of populations-spatial and environment relation | 32.0 | 49.5 |  |
| Sum of all eigenvalues |  |  | 8.87 |
| Sum of all canonical eigenvalues |  |  | 2.95 |

**Supplementary Table S4** Correlation coefficient between environmental factors and the first two ordination axes

|  | CCA1 | CCA2 | r^2^ | *p* |
| --- | --- | --- | --- | --- |
| Longitude | -0.9784 | -0.0653 | 0.97 | 0.001** |
| Latitude | 0.9078 | 0.1338 | 0.84 | 0.001** |
| Altitude | 0.2536 | -0.5152 | 0.28 | 0.035* |
| Temperature | 0.1597 | -0.5011 | 0.09 | 0.399 |
| Chlorophyll-*a* | -0.2568 | -0.2022 | 0.09 | 0.378 |
| TN | -0.2592 | -0.3809 | 0.09 | 0.398 |
| TP | -0.1811 | 0.2072 | 0.19 | 0.102 |

*, *p* <0.05；**, *p* <0.01; SPEC AX1: species axis 1; SPEC AX2: species axis 2; ENVI AX1: environmental factors axis 1; ENVI AX2: environmental factors axis 2;
